# Preoperative Diagnosis of Meckel’s Diverticulitis Mimicking Appendicitis in a Young Adult: A Case Report

**DOI:** 10.7759/cureus.88275

**Published:** 2025-07-18

**Authors:** Bernard L Hanekom, Rotimi Afolabi

**Affiliations:** 1 General Surgery, Kalgoorlie Hospital, Kalgoorlie, AUS; 2 Medicine, University of Western Australia, Kalgoorlie, AUS

**Keywords:** acute surgical abdomen, meckel’s diverticulitis, meckel's diverticulum, rare cause of acute abdominal pain, small bowel resection

## Abstract

Meckel’s diverticulitis is a rare but important differential in patients presenting with right iliac fossa pain and is often initially mistaken for acute appendicitis. We report a case of a 22-year-old male patient who presented with symptoms consistent with appendicitis but was found on preoperative computed tomography (CT) to have Meckel’s diverticulitis. He subsequently underwent surgical resection and made an uneventful recovery. This case highlights the diagnostic utility of CT in acute abdomen and the importance of considering Meckel’s diverticulitis within the differential diagnosis of right iliac fossa pain in adult patients.

## Introduction

Meckel’s diverticulum is the most common congenital malformation of the gastrointestinal tract, occurring in approximately 2% of the population [1]. It is a true diverticulum, containing all layers of the intestinal wall, and results from the incomplete obliteration of the vitellointestinal duct during fetal development. It is usually located within 60-100 cm of the ileocecal valve, and more than half contain ectopic tissue [1]. Most cases remain asymptomatic; however, complications occur in 4-6% of cases and include bleeding, bowel obstruction, inflammation (Meckel’s diverticulitis), fistulae, and tumors [2]. Clinical diagnosis of Meckel’s diverticulitis is challenging, as it typically presents similarly to more common causes of acute abdomen, such as appendicitis [3,4]. Diagnostic uncertainty in cases of Meckel’s diverticulitis can lead to management delays and increased morbidity [5].

## Case presentation

**Figure 1:**
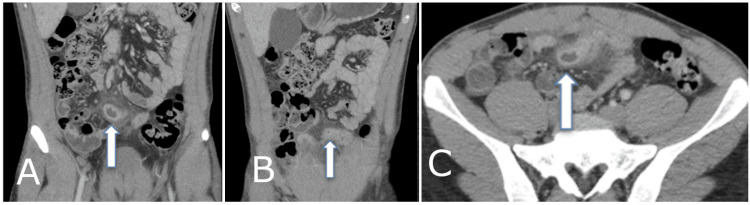
Coronal (A, B) and axial (C) views of computed tomography (CT) scan showing a blind-ending, inflamed tubular structure originating from the ileum (arrows).

A 22-year-old male patient presented to a rural emergency department reporting a one-day history of sudden onset, severe right iliac fossa pain exacerbated by movement. He reported associated nausea and subjective fevers but denied vomiting, bowel changes, or urinary symptoms. He had no family history of bowel disease and was otherwise healthy, without significant medical history or prior operations. On initial examination, he was afebrile and hemodynamically stable. The abdominal exam revealed a non-distended abdomen with localized peritonism in the right lower quadrant, without guarding or rebound tenderness elsewhere. Initial blood tests showed a white cell count of 13 x 10^9^/L (reference range: 4-11 x 10^9^/L) and a C-reactive protein (CRP) level of 8 mg/L (reference range: <5 mg/L). The rest of his laboratory panel and a urine dipstick were unremarkable. The patient's Alvarado score was 8, indicating a high likelihood of appendicitis. He proceeded to a computed tomography (CT) scan of his abdomen and pelvis, which demonstrated a blind-ending, inflamed tubular structure arising from the distal ileum, measuring 4 cm in length, consistent with an inflamed Meckel’s diverticulum (Figures 1A-1C). The appendix appeared normal.

**Figure 2:**
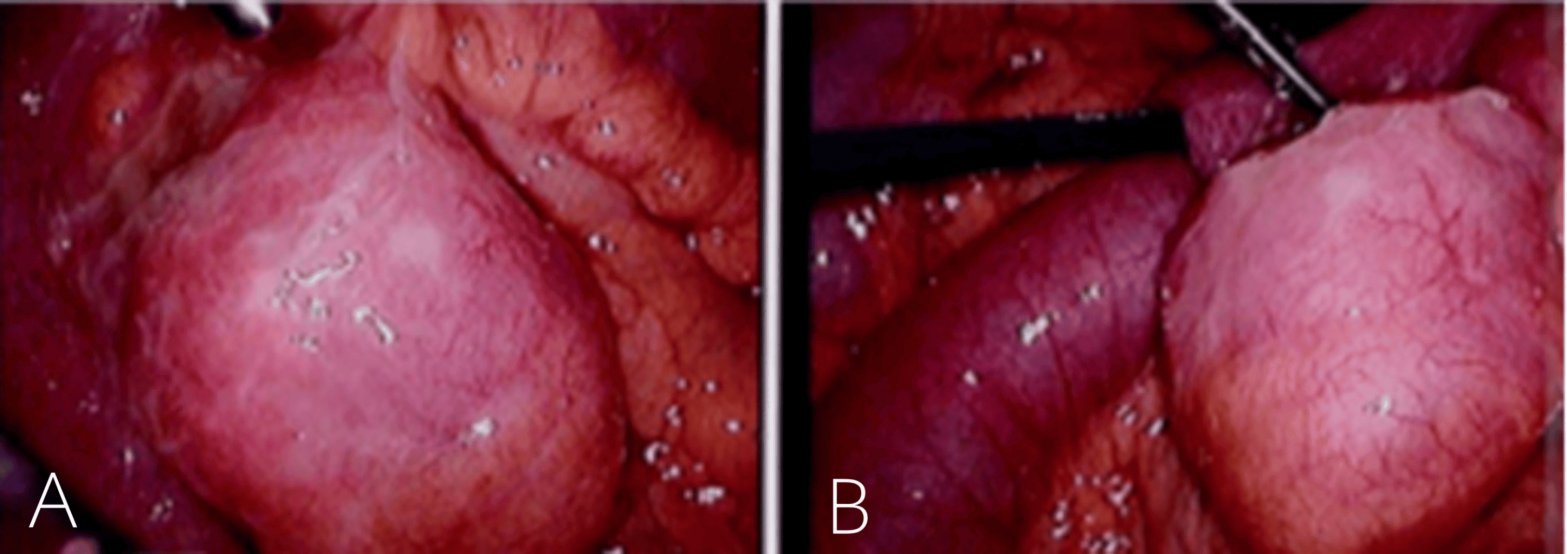
Two laparoscopic photos (A, B) taken intraoperatively showing an inflamed tubular structure arising from the ileum, consistent with the diagnosis of Meckel’s diverticulitis.

**Figure 3:**
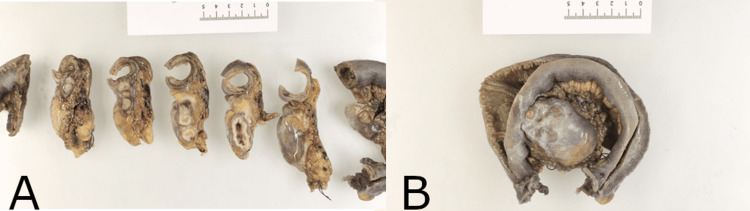
Macroscopic pathology specimens of surgical resection showing cross-section of Meckel’s diverticulum (A) and small bowel resection with Meckel’s diverticulum (B).

**Figure 4:**
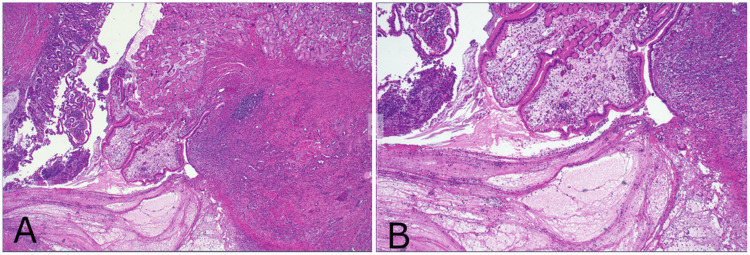
Histological specimens of Meckel’s diverticulum (A, B).

He underwent diagnostic laparoscopy, which confirmed the diagnosis (Figures 2A-2B), with conversion to a miniature midline laparotomy due to adhesions and technical considerations. A segmental small bowel resection of the Meckel’s diverticulum was performed, and a stapled functional end-to-end anastomosis was created. An incidental appendicectomy was also performed. Postoperatively, he remained on intravenous antibiotics before transitioning to oral antibiotics. He made an uneventful recovery and was discharged on postoperative day 4. The specimen was sent for histopathology (Figures 3A-3B), which confirmed an inflamed Meckel’s diverticulum with evidence of foveolar and oxyntic type gastric mucosa, ulceration, rupture, and peritonitis, without malignancy (Figures 4A-4B). The appendix was histologically normal.

## Discussion

Meckel’s diverticulum is present in up to 2% of the population and causes symptoms in only a small subset of patients, particularly pediatric patients [2,6]. Meckel’s diverticulitis is a rare cause of acute abdomen in adults and is typically indistinguishable from appendicitis preoperatively [7]. As a result, Meckel’s diverticulum is usually only diagnosed intraoperatively in most cases [2,4]. Complications of Meckel’s diverticulum include gastrointestinal bleeding (from ectopic gastric mucosa), small bowel obstruction, and inflammation [2]. In adults, Meckel’s diverticulitis is often diagnosed during surgical exploration for presumed appendicitis. However, the increasing use of CT for acute abdominal pain makes preoperative diagnosis more likely. CT is useful for identifying Meckel’s diverticulum and associated complications, and it rules out other common but serious causes of acute abdomen [8]. CT findings suggestive of Meckel’s diverticulitis include a blind-ending structure arising from the ileum with associated surrounding fat stranding and a normal appendix [8]. In our patient, the CT scan accurately identified Meckel’s diverticulitis preoperatively, which benefited preoperative patient counselling and improved surgical planning.

Ultrasound has limited use in diagnosing Meckel’s diverticulum, although it may have a role in assessing complications such as intra-abdominal abscess [2]. The technetium-99m pertechnetate scan (Meckel’s scan) is aimed at identifying ectopic gastric mucosa and is considered the most accurate non-invasive method for diagnosing Meckel’s diverticulum in pediatric patients [3]. The technetium-99m scan is more useful in pediatric patients with bleeding, who frequently have ectopic gastric mucosa, but is less useful in adults presenting with an inflamed diverticulum due to poor sensitivity and specificity [2,9,10]. Debate exists on whether asymptomatic, incidentally detected Meckel’s diverticulum requires resection, and often a selective approach is utilized to remove a 'high-risk' diverticulum [6]. There is general agreement that all symptomatic Meckel’s diverticula warrant surgical resection. Surgical approaches include simple diverticulectomy or segmental small bowel resection [2,6,11]. In our case, segmental resection was performed due to the degree of inflammation and to ensure complete removal. Prophylactic appendicectomy was performed to avoid future problems.

This case supports the expanding role of CT imaging in acute abdomen, particularly in equivocal cases or when there is concern for alternate pathology. As CT imaging becomes more readily available, the preoperative diagnosis of Meckel’s diverticulitis may become more common, allowing for improved surgical planning and potentially reducing negative appendicectomy rates.

## Conclusions

Meckel’s diverticulitis is a rare but important differential diagnosis for acute right iliac fossa pain. Meckel’s diverticulum can present as a wide range of symptoms, and Meckel’s diverticulitis is difficult to distinguish from other common causes of acute surgical abdomen on clinical grounds alone. Although it often mimics appendicitis, changes in practice and availability of cross-sectional imaging allow for a higher rate of preoperative diagnosis. Traditionally, an overwhelming majority of Meckel’s diverticulitis has been diagnosed intraoperatively. However, the rate of preoperative diagnosis will likely increase with current imaging practice. This case demonstrates the value of CT imaging in differentiating causes of acute abdomen and highlights the importance of maintaining a broad differential diagnosis in young adults with abdominal pain.
